# Causal Relationship Between Parathyroid Hormone and the Risk of Osteoarthritis: A Mendelian Randomization Study

**DOI:** 10.3389/fgene.2021.686939

**Published:** 2021-07-26

**Authors:** Guiwu Huang, Yanlin Zhong, Wenchang Li, Weiming Liao, Peihui Wu

**Affiliations:** Department of Joint Surgery, The First Affiliated Hospital of Sun Yat-sen University, Sun Yat-sen University, Guangzhou, China

**Keywords:** parathyroid hormone, osteoarthritis, Mendelian randomization analysis, genome-wide association studies, single-nucleotide polymorphisms

## Abstract

**Background:**

Previous studies have demonstrated an inverse association between parathyroid hormone (PTH) and the risk of osteoarthritis (OA). However, it remains unknown whether such association reflects causality. We aimed to apply a Mendelian randomization (MR) approach to investigate the causal association between PTH and OA.

**Materials and Methods:**

We performed a two-sample MR analysis using summary statistics from 13 cohorts (PTH, *N* = 29,155) and a recent genome-wide association study meta-analysis (OA, *N* = 455,221) by the UK Biobank and Arthritis Research UK OA Genetics (arcOGEN). MR analyses were carried out mainly using the inverse-variance-weighted method. Sensitivity analyses were performed to test the robustness of the associations using the weighted median method, the MR–Egger method, and “leave-one-out” analysis. Analyses were performed again to test whether the associations remained statistically significant after excluding any outlier variants that were detected using the MR-PRESSO (Mendelian Randomization Pleiotropy RESidual Sum and Outlier) test.

**Results:**

Five single-nucleotide polymorphisms (SNPs) were selected as instrumental variables at the genome-wide significance threshold (*p* < 5 × 10^–8^). The causal effect between PTH and OA was genetically predicted using the inverse-variance-weighted method (odds ratio = 0.67, 95% confidence interval: 0.50–0.90; *p* = 0.008). This result was borne out using the weighted median method (odds ratio = 0.73, 95% confidence interval: 0.60–0.90; *p* = 0.004). The causality remained robust after discarding the outlier variants as well as SNPs associated with confounding factors.

**Conclusion:**

MR analysis supported a potential causative relationship between decreased serum circulating PTH and a higher risk of hip and knee OA.

## Introduction

Osteoarthritis (OA) is a common, chronic, and disabling disease that is characterized by cartilage degradation, osteophyte formation, joint inflammation, and loss of normal joint function, making it one of the top 10 debilitating diseases in the 21st century (O’Neill et al., 2018; Runhaar and Zhang, 2018; Bierma-Zeinstra et al., 2020). The prevalence and incidence of OA have increased rapidly in the past few years (Biver et al., 2019; Sebbag et al., 2019; Abramoff and Caldera, 2020). A report by the World Health Organization in 2013 (Tong, 2013) suggested that nearly 130 million people will be diagnosed with OA by 2050, which will generate an estimated national cost of $45 billion in the United States (Zhao et al., 2019).

Although the exact cause of OA remains unclear, research has demonstrated that advanced age, obesity, injury, and gut microbiota composition are all catalysts that speed up the process of OA (Vina and Kwoh, 2018; Zengini et al., 2018; Biver et al., 2019). In addition, previous biomedical studies have reported an association between parathyroid hormone (PTH) deficiency and the risk of OA (Emdin et al., 2017; Shao et al., 2020). PTH is a polypeptide of 84 amino acids that is secreted by the parathyroid gland; its function is to maintain normal extracellular calcium levels (Martin, 2016). In addition, PTH exerts an anabolic effect on bone (Goltzman, 2018). Some studies have reported that the application of PTH alleviates OA progression (Lugo et al., 2012; Chen et al., 2018; Shao et al., 2020), possibly because PTH also has anti-senescence and anti-inflammatory functions (Platas et al., 2017). Despite the fact that PTH concentration is related to OA progression (Wysolmerski, 2012; Wojda and Donahue, 2018), very little is known about the effects of individual single-nucleotide polymorphisms (SNPs). To date, biomedical and observational studies have failed to perform a complete investigation of these effects because of the sophistication and great expense of the required methods (de la Torre Hernández and Edelman, 2017).

Mendelian randomization (MR) is considered as a reliable approach for evaluating the causal effect between a risk factor and a clinically relevant outcome. This technique can overcome common pitfalls of traditional studies, such as confounding factors and reverse causation (Emdin et al., 2017; Zheng et al., 2017). Consequently, the objective of this study was to determine the potential causal effect of PTH on the risk of OA using MR analysis.

## Materials and Methods

### Study Overview

**FIGURE 1:**
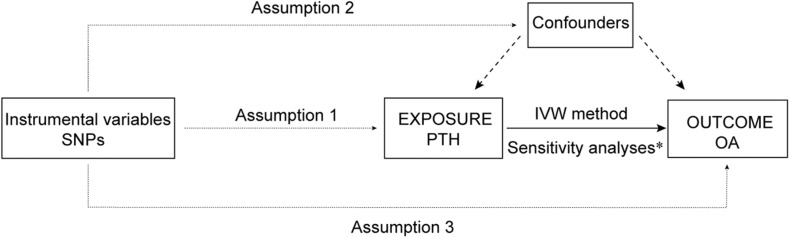
An overview of the study design. SNP, single-nucleotide polymorphism; PTH, parathyroid hormone; OA, osteoarthritis; IVW, inverse-variance weighted; IV: instrumental variable. Assumption 1, the genetic variants selected as IVs should be strongly associated with the risk factor of interest; Assumption 2, the genetic variants used as IVs should not be associated with any confounders; and Assumption 3, the IVs should affect the risk of the outcome merely through the risk factor, not *via* any alternative pathways. *Sensitivity analyses: weight median method, MR–Egger regression, MR-PRESSO, and leave-one-out test.

This study applied MR analysis to determine whether PTH is causally associated with OA using summary data of SNP exposure PTH and SNP outcome OA. An overview of the study design is shown in Figure 1.

### GWAS Summary Statistics

To identify putative effector genes for the risk of OA, a meta-analysis of genome-wide association studies (GWAS) was carried out across 17.5 million single-nucleotide variants. Up to 455,221 individuals from a European population (including 77,052 cases and 378,169 controls) included in the UK Biobank and Arthritis Research UK OA Genetics (arcOGEN) resources were included (Tachmazidou et al., 2019). The GWAS of UK Biobank was adjusted by the first 10 PCs (principle components, computed using fast PCA (Galinsky et al., 2016) and high-quality directly typed markers from the unrelated set of Europeans), sex, age at recruitment, and genotyping chip. Moreover, the GWAS of arcOGEN was adjusted by the first 10 PCs. The detailed diagnostic information and the numbers for cases and controls in the GWAS for each OA phenotype are shown in the Supplementary Appendix Note. The relevant ethics committees approved all studies that contributed data to these analyses, and all participants provided written informed consent.

### Selection of Instrumental Variables

The genetic instrumental variables associated with serum circulating PTH concentrations were obtained from a GWAS meta-analysis that comprised 29,155 participants of European ancestry from 13 quality-controlled, cohort-level results files (*N* = 22,653 and *N* = 6,502 in the discovery and replication analyses of European ancestry, respectively) (Robinson-Cohen et al., 2017). All cohort-level analyses were restricted to European-ancestry individuals who passed the quality control of the cohort. All instrumental variables were independent at the genome-wide significance level (*p* < 5 × 10^–8^) with linkage disequilibrium (LD) *r*^2^< 0.01, which validated the independence of the selected genetic variants.

We then calculated the *F* statistic for each of the SNPs using the following formula: *R*^2^×(*N*−2)/(1−*R*^2^). Here, *R*^2^ indicates the proportion of variance in PTH explained by a given SNP, and *N* indicates the sample size. More specifically, *R*^2^ was calculated using the following formula: *R*^2^=[2×Beta^2^×(1−*EAF*)×EAF]/[2×Beta^2^×(1−*EAF*)×EAF2×SE^2^×*N*×(1−*EAF*)×EAF]. Here, beta indicates the genetic effect of the SNP on PTH, EAF is the effect allele frequency, SE is the standard error, and *N* is the sample size. It is recommended that *F* > 10 to avoid employing weak genetic instruments.

Palindromic SNP refers to the sequence of bases in the same order on the positive and negative strands of DNA, but in the opposite direction. Moreover, when EAF > 0.42 (intermediate allele frequencies), it was difficult to determine whether the SNP was on the positive and negative strands of DNA; thus, harmonization could not be carried out. In the harmonizing process, SNPs were excluded if they were non-concordant or palindromic with intermediate allele frequencies.

### Statistical Analyses

The “Two-Sample-MR” package (version 0.5.5) in R software version 4.0.2^1^ was used to perform the statistical analyses; *p* < 0.05 was the threshold for a significant difference. All estimates were reported with two-tailed *p*-values. Potential causality between PTH and OA was estimated using the inverse-variance-weighted (IVW) and weighted median methods. The estimate of each SNP was conducted by single SNP analysis. Moreover, the power to estimate a causal risk ratio of disease at a two-side α of 0.05 and β of 80% was calculated with an online power calculation^2^ in MR studies with binary outcomes.

Cochran’s Q statistic was calculated as a measure of potential heterogeneity. The intercept derived from MR–Egger regression was also calculated to provide valid causal estimates of directional pleiotropy. We also performed “leave-one-out” analysis to evaluate whether the observed causal relationship was reliant on any single SNP. Finally, the MR-PRESSO (Mendelian Randomization Pleiotropy RESidual Sum and Outlier) test was conducted to detect any outliers with potential pleiotropy. Once outliers were identified, they were removed from the data and the MR analysis was repeated.

Confounder-related SNPs refer to SNPs that are related to the outcome (OA in our study) or its risk factors except the selected exposure (PTH in our study). As shown in Table 1, in this study, we retrieved previously published OA-related MR studies from PubMed and identified causally associated risk factors with OA. These risk factors might be the potential confounding factors of this MR study. Therefore, we further conducted a comprehensive search in the GWAS Catalog^3^ (accessed on November 17, 2020) for whether any SNP in this study was associated with these confounders at the genome-wide significance of *p* < 5 × 10^–8^. Analyses were then performed again to test whether the associations remained significant after excluding SNPs associated with traits other than PTH.

**TABLE 1:** Previously published OA-related MR studies from PubMed and identified causally associated risk factors with OA.

Trait(s)	Causally associated risk factors
Physical index	body mass index, femoral neck bone mineral density, and systolic blood pressure
Serum components	sex hormone-binding globulin, insulin-like growth factor-1, adipokines, LDL-C, low-density lipoprotein cholesterol, C-reactive protein, zinc, and copper status
Lifestyle	coffee consumption, smoking, and education

## Results

Six lead SNPs were identified at the genome-wide significance threshold (*p* < 5 × 10^–8^), which explained 4.5% of the variance in circulating PTH (Robinson-Cohen et al., 2017). In order to validate the independence of the selected genetic variants, the LD test was performed in the LDlink website^4^, which revealed a slight disequilibrium linkage between rs6127099 and rs35194449 (LD *r*^2^ = 0.38). Rs6127099 was selected as instrumental variable with *p* = 2.40 × 10^–72^, while rs35194449 was selected with *p* = 1.8 × 10^–10^. Therefore, rs35194449 with a larger *p* value was excluded from instrumental variables. Eventually, five SNPs were used as instrumental variables for PTH in the present study (Table 2). *F* statistic in the regression model was using to evaluate the strength of the association between instrumental variables and exposure factor (PTH in this study). Moreover, all the *F* statistics are more than 10, verifying that the instrumental variables were strongly associated with exposure factor (PTH in this study) (Zheng et al., 2017). It was widely considered that the instrumental variables had sufficient strength, and the results had high credibility (Burgess and Thompson, 2011). No SNPs were excluded in the harmonizing process.

**TABLE 2:** Characteristics of SNPs for serum PTH levels from the GWAS meta-analysis.

SNP	Gene	EA	OA	EAF	Beta-Exp	Se-Exp	*P* val-Exp	Beta-Out	Se-Out	*P* val-out	F-statistics
rs6127099	CYP24A1	T	A	0.34	0.07	0.003	2.40 × 10^–72^	–0.0162	0.0086	0.06143	206
rs4074995	RGS14	G	A	0.71	0.03	0.003	3.30 × 10^–23^	–0.016	0.0086	0.06259	127
rs219779	CLDN14	G	A	0.75	0.04	0.003	8.90 × 10^–22^	–0.0157	0.0086	0.0679	59
rs4443100	RTDR1	G	C	0.32	0.02	0.003	4.10 × 10^–11^	–0.0224	0.0083	0.006762	74
rs73186030	CASR	T	C	0.14	0.03	0.003	1.20 × 10^–9^	–0.0382	0.0114	0.000774	768

*Gene, the nearest gene to PTH-associated SNP; Beta, the per-allele effect on serum PTH levels; *p* val, the *p*-value for the genetic association; Exp, exposure, PTH in this study; out, outcome osteoarthritis in this study. PTH, parathyroid hormone; GWAS, genome-wide association study; SNP, single-nucleotide polymorphism; EA, effect allele; OA, other allele; EAF, effect allele frequency; SE, standard error.*

**FIGURE 2:**
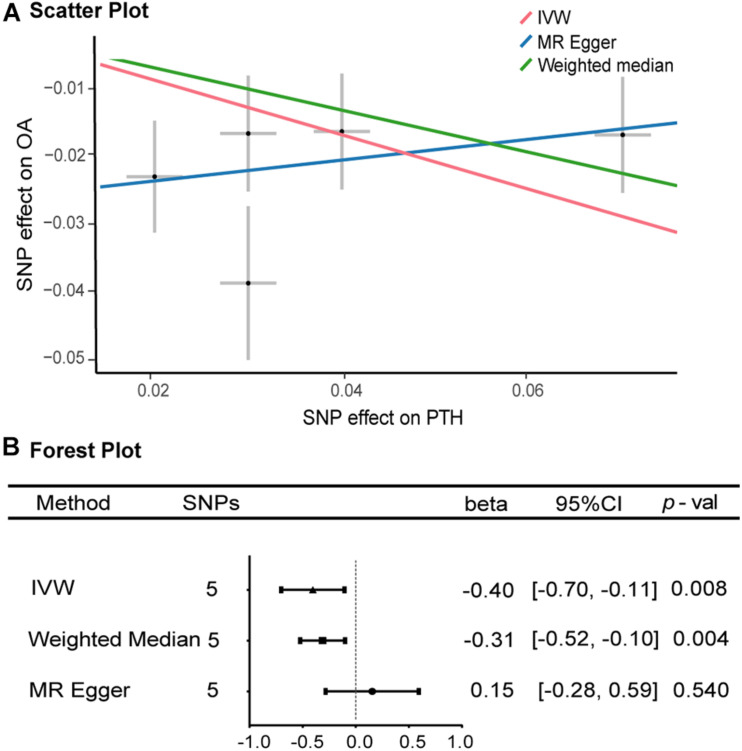
Scatter and forest plots of the Mendelian randomization analyses for the association of parathyroid hormone with the risk of osteoarthritis. **(A)** Scatter plot. **(B)** Forest plot. OR, odds ratio, CI, confidence interval; IVW, inverse-variance-weighted method; MR, Mendelian randomization; SNP, single-nucleotide polymorphism. **(B)** The *X*-axis scale is ln (OR), and beta = ln (OR).

**FIGURE 3:**
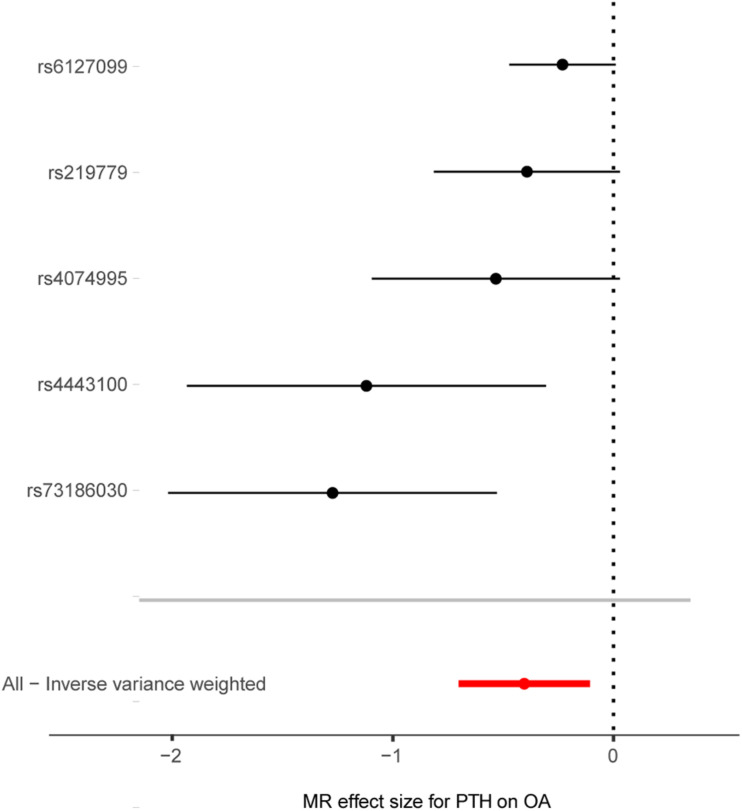
Forest plot of the causal effects of PTH-associated single-nucleotide polymorphisms on osteoarthritis. MR, Mendelian randomization; PTH, parathyroid hormone, OA, osteoarthritis, OR, odds ratio. The *X*-axis scale is ln (OR), and beta = ln (OR).

As shown in Figures 2, 3, along with the five stringently selected SNPs for the subsequent two-sample MR analysis, there was strong evidence to support a causal association between PTH and OA using the IVW method (odds ratio [OR] = 0.67, 95% confidence interval [CI]: 0.50–0.90; *p* = 0.008). Given 80% power, we found that the detectable odds ratio was 0.948; therefore, this study was powered enough to detect larger effect sizes (smaller ORs) than 0.948. The associations were consistent with the sensitivity analyses using the weighted median method (OR = 0.73, 95% CI: 0.60–0.90; *p* = 0.004). The effect was only slightly attenuated using the MR–Egger method, yielding a null causal estimation between PTH and OA (OR = 1.17; 95% CI: 0.75–1.81; *p* = 0.540), although the direction of the association was somewhat reversed. Considering that the weighted median method is advantageous because it retains greater precision of the estimates compared with MR–Egger analysis (Bowden et al., 2016), the results of the MR analysis support an inversely causative relationship between PTH and OA. Furthermore, no outliers were detected with potential pleiotropy using the MR-PRESSO method. Moreover, the *p*-value for the MR-PRESSO Global test was 0.027, which suggested a potential sign of heterogeneity.

**FIGURE 4:**
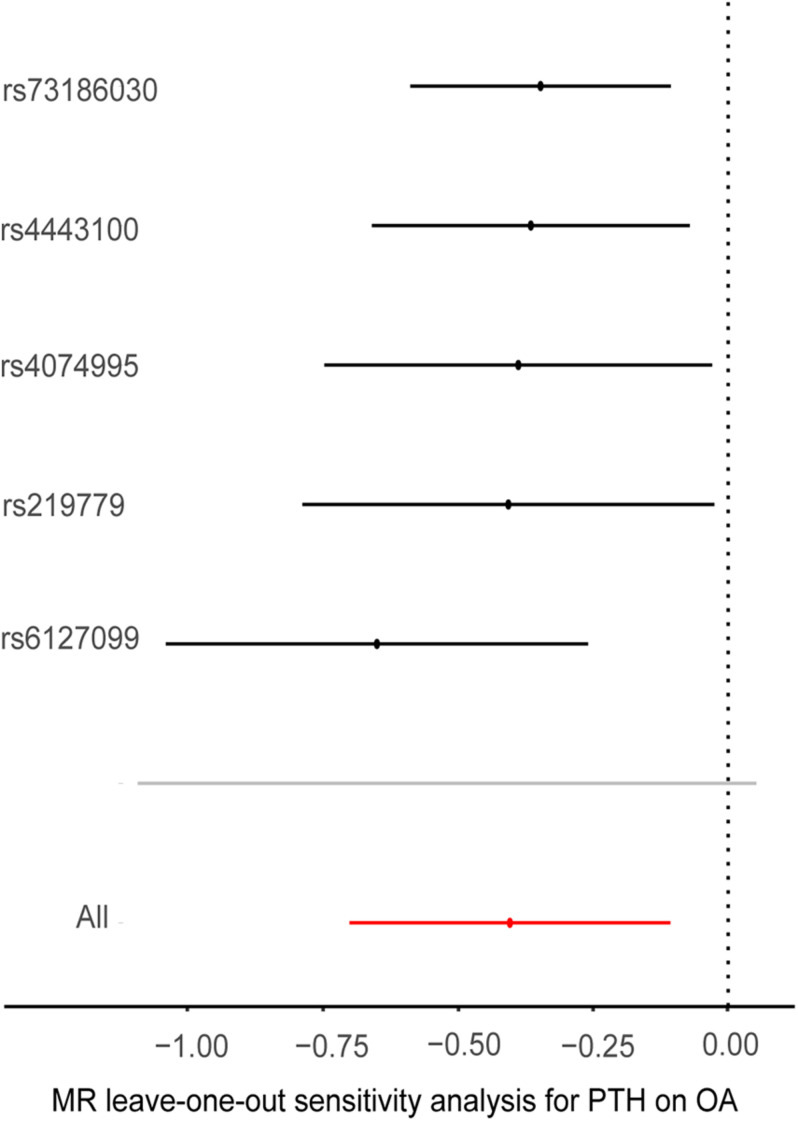
Plots of “leave-one-out” analyses for Mendelian randomization analyses of the causal effects of parathyroid hormone with the risk of osteoarthritis. MR, Mendelian randomization; PTH, parathyroid hormone, OA, osteoarthritis, OR, odds ratio. The *X*-axis scale is ln (OR), and beta = ln (OR).

Cochran’s Q statistic suggested a potential sign of heterogeneity: Q value (*df*) = 10.390 (4), *p* = 0.03. However, there was no indication of pleiotropy using the intercept derived from the MR–Egger regression (Egger intercept = −0.026, *p* = 0.07). As shown in Figure 4, the results of the leave-one-out analysis revealed that there were no potentially influential SNPs driving the causal link between PTH and OA in the replication analysis.

As shown in Table 3, we found one SNP (rs4074995) that is associated with potential confounders (calcium). As for the other SNPs, we did not find these SNPs associated with any confounding factors. Therefore, we only selected calcium as a confounder in our study. After excluding this SNP (rs4074995) associated with potential confounders, results from the statistical analysis remained essentially consistent (OR = 0.68, 95% CI: 0.47–0.97, *p* = 0.03, using the IVW method).

**TABLE 3:** The reported traits of selected SNP searched in GWAS catalog.

SNP	Trait(s)
rs6127099	Vitamin D, parathyroid hormone, creatinine, and glomerular filtration rate
rs4074995	Calcium, parathyroid hormone
rs219779	Parathyroid hormone
rs4443100	Parathyroid hormone
rs73186030	Parathyroid hormone

*SNP, single-nucleotide polymorphism; GWAS, genome-wide association study.*

## Discussion

This is the first MR study to examine whether the serum PTH concentration is causally associated with risk of OA. Our study based on genetics provides evidence that a lower serum PTH concentration is causally associated with an increased risk of OA.

OA is a degenerative disease that involves the subchondral bone, the bone plate, and the cartilage. The abnormal bone cell metabolism, subchondral bone remodeling, and cartilage degradation and loss are involved in the pathogenesis of OA (Lajeunesse, 2004). OA develops gradually overtime. The known risk factors for OA include genetic predisposition, being overweight, previous joint injury, increased age, and also improper joint activity incurred from occupation or hobbies (Ng et al., 2016).

In the present study, we used MR analysis to provide evidence to support a causal association between serum PTH level and the risk of OA. Previous animal experiment revealed definitely curative effects that PTH treatment and recombinant teriparatide treatment are beneficial in the management and prevention of OA, osteoporosis, and bone fracturing. PTH contributes to increasing the activity and number of osteoclasts, maintaining calcium homeostasis, and increasing bone mineral density (BMD). What is more, it is also responsible for alleviating subchondral osteosclerosis, improving articular cartilage surface architecture, and thereby repairing cartilage damage (Orth et al., 2013). In the early stage of OA, PTH may suppress the loss of GAG and Col II that occurred in OA cartilage and suppress the expression of Col X in articular chondrocytes caused by OA induction (Eswaramoorthy et al., 2012). Additionally, one study reported that an OA rat model had the ability to bear more weight and run for a longer time after PTH (1–34) treatment (Chen et al., 2018). These improvements were likely caused by reduced chondrocyte terminal differentiation and apoptosis as well as increased autophagy. Furthermore, PTH may inhibit p16^*ink4a*^, a cyclin-dependent kinase inhibitor and senescence biomarker, resulting in the decreased accumulation of senescent cells in subchondral bone and an improved bone marrow microenvironment to stimulate the process of bone remodeling (Cui et al., 2020). However, there has been little human data on this issue, with a cross-sectional study including 5,880 Korean participants who demonstrated that although there was a trend for a negative association in women, no statistically significant association was found between endogenous PTH and knee OA (Lee, 2016). In order to evaluate the therapeutic outcome of PTH as a disease-modifying therapy in OA, more human data are required.

Another MR analyses conducted by Qu et al. (2020) showed that serum calcium levels were inversely associated with overall OA [OR 0.712, 95% CI (0.595, 0.850)]. According to a 48-month follow-up study, frequent milk consumption may be associated with reduced OA progression in women partially through elevated dietary calcium intake (Lu et al., 2014). A cross-sectional study conducted by Li et al. (2016) with 2,855 Chinese subjects demonstrated that an inverse association existed between serum calcium concentration and radiographic knee OA. Furthermore, an *in vitro* study also demonstrated the effect of calcium/calmodulin-dependent signaling on the intracellular transduction of bovine articular chondrocytes. Thus, the effect of serum PTH concentration on OA may be mediated by serum calcium. However, the potential biological mechanisms about how the elevation of serum PTH concentration decreases the risk of OA were rarely reported and further researches are needed to uncover the pathways.

Using the MR framework, a causative association between PTH and the risk of OA was fully explored. The credibility of this study is demonstrated by the use of data from large sample sizes. Data from over 400,000 European individuals (OA: *N* = 455,221; PTH: *N* = 29,115) from the latest GWAS meta-analysis were used in this study. The results from the IVW (OR = 0.67, *p* = 0.008) and weighted median (OR = 0.73, *p* = 0.004) analyses suggested an inverse causative association between PTH and OA. In contrast, the MR–Egger approach indicated that there were no causative associations between PTH and OA (OR = 1.17, *p* = 0.540). The gene exposure (genetically serum PTH levels) using the MR–egger method did not correlate with the bias caused by directional pleiotropy, which reduced overestimation of a causal effect due to pleiotropy but at the cost of lower power. It did not provide additional evidence for causal inference in the MR–Egger analysis when directional pleiotropy was not detected (Burgess and Thompson, 2017). Meanwhile, the estimation (both the intercept and the slope) by MR–Egger with only five instrumental variables is not stable enough. Thus, the IVW and weighted median methods appear to be superior to the MR–Egger test, and we have therefore used the results from these methods to support our conclusions.

There are several limitations to this study. The summary GWAS data were restricted to individuals of European descent; thus, the findings might be biased and not applicable to other populations. Future studies in other non-European populations will provide a more comprehensive understanding. Meanwhile, we failed to explore any potential nonlinear relationships or stratification effects as a result of using summary data. Moreover, potential sample overlap might be another source of bias in these MR analyses. However, the F statistics calculated in the sensitivity analysis were all large enough, suggesting that the bias might be minimal. Despite of these potential limitations, sensitivity analyses to test the influence of any individual SNPs revealed that the causal estimates were robust, thus suggesting that this study accurately represents the close association between PTH deficiency and risk of OA.

## Conclusion

The MR analysis instructed a potential causal association between increased serum PTH concentration and a lower OA risk. Current findings might offer a chance to search out the mechanisms of serum PTH concentration on the OA progression.

## Data Availability Statement

The original contributions presented in the study are included in the article/Supplementary Material, further inquiries can be directed to the corresponding author.

## Author Contributions

GH and YZ collected the data, conducted the MR analysis, and wrote the manuscript. PW and GH contributed to the conceptualization, methodology, data acquisition and curation, formal analysis, visualization, writing, and editing. WCL and WML contributed to the methodology, interpretation of data, writing, and editing. All authors reviewed the manuscript.

## Conflict of Interest

The authors declare that the research was conducted in the absence of any commercial or financial relationships that could be construed as a potential conflict of interest.

## Publisher’s Note

All claims expressed in this article are solely those of the authors and do not necessarily represent those of their affiliated organizations, or those of the publisher, the editors and the reviewers. Any product that may be evaluated in this article, or claim that may be made by its manufacturer, is not guaranteed or endorsed by the publisher.

## Footnotes

1
https://www.r-project.org/

2
https://shiny.cnsgenomics.com/mRnd/

3
http://www.ebi.ac.uk/gwas

4
https://ldlink.nci.nih.gov/

## Abbreviations

CI: confidence interval
GWAS: genome-wide association studies
IVW: inverse-variance weighted
MR: Mendelian randomization
OA: osteoarthritis
PTH: parathyroid hormone
SNPs: single-nucleotide polymorphisms.
