# Supplementary material for: Causal Relationship Between Parathyroid Hormone and the Risk of Osteoarthritis: A Mendelian Randomization Study
Source: Front Genet. 2021 Jul 26;12:686939. doi: 10.3389/fgene.2021.686939 (PMC8352559; doi:10.3389/fgene.2021.686939)
Supplement: Supplementary file 1 [file Data_Sheet_1.docx]

**Causal Relationship Between Parathyroid Hormone and** **the Risk of** **Osteoarthritis: a Mendelian Randomization Study**

Guiwu Huang^1#^, Yanlin Zhong^1#^, Wenchang Li^1^, Weiming Liao^1^, Peihui Wu^1^*

^1^Department of Joint Surgery, The First Affiliated Hospital of Sun Yat-sen University, Sun Yat-sen University, Guangzhou, China.

^#^These authors have contributed equally to this work

*Corresponding author: Peihui Wu, Department of Joint Surgery, The First Affiliated Hospital of Sun Yat-sen University, Sun Yat-sen University, Guangzhou, China, Email: [wupeihui3@mail.sysu.edu.cn](mailto:wupeihui3@mail.sysu.edu.cn), Telephone: +86-13332807351

Appendix note

Up to 455,221 individuals (77,052 cases and 378,169 controls) from a European population included in the UK Biobank and Arthritis Research UK OA Genetics (arcOGEN) resources were included^[1]^. Detailed diagnostic information and the numbers of cases and controls in GWAS for each osteoarthritis phenotype were as follow:

**UK Biobank** (70,532 cases and 369,983 controls): The self-reported status established during interview with a nurse (Initial assessment visit) and the Hospital Episode Statistics ICD10 primary and secondary codes were used to define osteoarthritis cases. Four osteoarthritis phenotypes were included: self-reported or hospital-diagnosed osteoarthritis at any site based on ICD10 hospital record codes M15-M19 (n=70,532); hospital-diagnosed hip osteoarthritis based on ICD10 hospital record M16 (n=12,850); hospital-diagnosed knee osteoarthritis based on ICD10 hospital record M17 codes (n=21,921); and hospital-diagnosed hip and/or knee osteoarthritis (M16 or M17; n=32,907). And from the controls, individuals with primary or secondary ICD10 codes M05 through M14 were excluded, as well as arthrosis codes M15-M19 to minimise misclassification in the control datasets to the extent possible. In all analyses, individuals with inflammatory poly-arthropathies were excluded. As a result, 369,983 individuals were selected as control samples.

**Arthritis Research UK Osteoarthritis Genetics (arcOGEN)** (6,520 cases): arcOGEN is a collection of unrelated, UK-based individuals of European ancestry with knee and/or hip osteoarthritis from the arcOGEN Consortium^[2,3]^. Based on clinical evidence of disease to a level requiring joint replacement or radiographic evidence of disease (Kellgren–Lawrence grade ≥2), 6,520 cases comprised three osteoarthritis phenotypes were ascertained: hip osteoarthritis (2,854 cases), knee osteoarthritis (3,034 cases), and hip and/or knee osteoarthritis (6,520 cases). And 8,186 controls were selected from United Kingdom Household Longitudinal Study (UKHLS), which was a longitudinal panel survey of 40,000 UK household representative of the UK population.

[1] Tachmazidou I, Hatzikotoulas K, Southam L*, et al.* Identification of new therapeutic targets for osteoarthritis through genome-wide analyses of UK Biobank data[J]. Nat Genet, 2019, 51(2): 230-236.

[2] Panoutsopoulou K, Southam L, Elliott KS*, et al.* Insights into the genetic architecture of osteoarthritis from stage 1 of the arcOGEN study[J]. Ann Rheum Dis, 2011, 70(5): 864-867.

[3] Zeggini E, Panoutsopoulou K, Southam L*, et al.* Identification of new susceptibility loci for osteoarthritis (arcOGEN): a genome-wide association study[J]. Lancet, 2012, 380(9844): 815-823.
